# Protection of retinal function and morphology in MNU-induced retinitis pigmentosa rats by ALDH2: an in-vivo study

**DOI:** 10.1186/s12886-020-1330-8

**Published:** 2020-02-18

**Authors:** Weiming Yan, Pan Long, Dongyu Wei, Weihua Yan, Xiangrong Zheng, Guocang Chen, Jiancong Wang, Zuoming Zhang, Tao Chen, Meizhu Chen

**Affiliations:** 1 Department of Ophthalmology, The 900th Hospital of Joint Logistic Support Force, PLA (Clinical Medical College of Fujian Medical University, Dongfang Hospital Affiliated to Xiamen University), Fuzhou, 350025 China; 2 Center of Clinical Aerospace Medicine, Fourth Military Medical University, Xi’an, 710032 China; 3 Department of Ophthalmology, The West General Hospital of Chinese PLA, Chendu, 610083 China; 4 Tong’an No.1 High School of Fujian Province, Xiamen, 361100 China; 5 BeiJing HealthOLight Technology Co. Ltd, Beijing, 10010 China

**Keywords:** ALDH2, Retinitis pigmentosa, Electroretinogram, Photoreceptor degeneration, SIRT1, Endoplasmic reticulum stress

## Abstract

**Background:**

Retinitis pigmentosa (RP) is a kind of inherited retinal degenerative diseases characterized by the progressive loss of photoreceptors. RP has been a conundrum without satisfactory countermeasures in clinic until now. Acetaldehyde dehydrogenase 2 (ALDH2), a major enzyme involved in aldehyde detoxification, has been demonstrated to be beneficial for a growing number of human diseases, such as cardiovascular dysfunction, diabetes mellitus and neurodegeneration. However, its protective effect against RP remains unknown. Our study explored the impact of ALDH2 on retinal function and structure in N-methyl-N-nitrosourea (MNU)-induced RP rats.

**Methods:**

Rats were gavaged with 5 mg/kg Alda-1, an ALDH2 agonist, 5 days before and 3 days after MNU administration. Assessments of retinal function and morphology as well as measurement of specific proteins expression level were conducted.

**Results:**

Electroretinogram recordings showed that Alda-1 administration alleviated the decrease in amplitude caused by MNU, rendering protection of retinal function. Mitigation of photoreceptor degeneration in MNU-treated retinas was observed by optical coherence tomography and retinal histological examination. In addition, Western blotting results revealed that ALDH2 protein expression level was upregulatedwith increased expression of SIRT1 protein after the Alda-1 intervention. Besides, endoplasmic reticulum stress (ERS) was reduced according to the significant downregulation of GRP78 protein, while apoptosis was ameliorated as shown by the decreased expression of PARP1 protein.

**Conclusions:**

Together, our data demonstrated that ALDH2 could provide preservation of retinal function and morphology against MNU-induced RP, with the underlying mechanism at least partly related to the modulation of SIRT1, ERS and apoptosis.

## Background

Retinitis pigmentosa (RP) is an inherited retinal degenerative disease with the progressive loss of photoreceptors, which leads to the deterioration of retinal function and morphology [1]. Mutation in specific genes that encode proteins participating in visual phototransduction is the primary cause of the death of photoreceptors. The inheritance modes for RP include autosomal dominant, autosomal recessive, and X-linked subtypes. Until now, more than 3000 genetic mutations from approximately 70 genes have been linked to RP [2]. Although gene therapy and stem cell replacement of photoreceptors have shown great potential in the treatment of this kind of retinal degeneration, the overall outcome has been far from satisfactory due to the high heterogeneity of RP [3–5]. New and effective countermeasures to pharmacologically mitigate or rescue photoreceptor death are continuously and urgently required.

Aldehydes are highly cytotoxic and mutagenic molecules that could easily form adducts with macromolecules, such as proteins, DNA, and lipids. These adducts could disrupt normal physiological function. The accumulation of excessive aldehydes, namely, the aldehydic overload, is closely related to a number of pathologies. Except for those found ubiquitously in the environment, endogenous aldehydes are formed as a part of the normal metabolism of nucleotides, amino acids and lipids [6]. Aldehyde dehydrogenase 2 (ALDH2) is a typical enzyme responsible for aldehyde detoxification. It has long been known as the primary oxidative enzyme in ethanol metabolism, which could convert acetaldehydes, a subtype of aldehydes, to acetic acid predominantly in the liver [7]. In addition, ALDH2 could react with other kinds of aldehydes. In particular, ALDH2 plays a key role in oxidizing endogenous aldehydic products, such as malonyldialdehyde dimetyl 1,1,3,3 tetrametoxypropanol (MDA), and 4-hydroxy-2-nonenal (4-HNE), that arise from oxidative stress-induced lipid peroxidation (LPO). LPO is a chain reaction leading to the disruption of polyunsaturated fatty acids and forming the carbonyl derivatives, especially aldehydes [6]. Recently, ALDH2 has gained increasing attention for providing endogenous protection against several human diseases, including cardiovascular dysfunction, diabetes mellitus and neurodegenerative disorders [8]. The protective effect of ALDH2 has been attributed primarily to the detoxification capacity of aldehydes, although the exact molecular mechanisms have not been clearly elucidated. Specifically, Wang et al. reported that ALDH2 serves as an indispensable factor against cardiac anomalies with a possible mechanism related to autophagy regulation and facilitation of SUV39H-silent information regulator-1 (SIRT1)-dependent PGC-1alpha deacetylation in a mouse model of high-fat diet-induced obesity [9]. By clinical investigation and in an in-vitro rat model of atherosclerosis in smooth muscle cells, Yang et al. found that ALDH2 may slow the progression of atherosclerosis through a mechanism related to the attenuation of endoplasmic reticulum stress (ERS) and apoptosis [10]. By enhancing ALDH2 expression, Yu et al. demonstrated that the occurrence of myocardial fibrosis in diabetic rats was reduced, which mechanism may be related to the downregulation of the c-Jun NH2-terminal kinase (JNK) pathway [11].

The retina is a part of the central nervous system with a high supply of oxygen. It has always been continuously exposure to light stimulation, subjecting itself vulnerable to oxidative stress damage [12]. Inside the retina, photoreceptors contain a large number of mitochondria, which are highly membranous. In addition, the outer segments of the rod photoreceptors are consist of membranes that have an extremely high proportion of polyunsaturated lipids [13]. These membranous morphologies of retina, plus the frequent formation of reactive oxygen species (ROS) insides, make it extremely susceptible to LPO [14]. Indeed, ROS-induced LPO has been demonstrated to occur in various retinal diseases, including diabetic retinopathy(DR), age-related macular degeneration (AMD) and RP [15–17].

In terms of the reported ALDH2-induced protection against aldehyde-related diseases, we speculated that ALDH2 might also be able to alleviate retinal diseases [18]. Alda-1 (N-[1,3-benzodioxol-5-ylmethyl]-2,6-dichlorobenzamide) is a ALDH2 activator, which specifically increases the expression of ALDH2 and its enzymatic activity [6]. In fact, we previously demonstrated that pharmacological activation of ALDH2 by Alda-1 gavage upregulated the expression level of ALDH2 in the retina of DR rats and alleviated the retinal damage [19]. However, whether ALDH2 can preserve retinal function and morphology in RP remains unknown. Therefore, the present study aimed to investigate whether photoreceptor degeneration in a pharmacologically induced RP model could be alleviated by ALDH2. Furthermore, some possible mechanisms underlying the ALDH2-mediated effects against RP were explored. All these work may provide new insights into the exploration of new interventions against RP.

## Methods

### Animals and agent preparation

Sprague-Dawley (SD) rats (males, 6–8 weeks old) were obtained from the Laboratory Animal Center of the Fourth Military Medical University (License number: 2014270138S, Xi’an, China) and were maintained under standard laboratory conditions (room temperature of 18–23 °C, 40–65% humidity, 12 h dark/light cycle), with food and water available ad libitum. All animal housing and handling conditions abided by the Association for Research in Vision and Ophthalmology Statement (ARVO) for the use of animals in Ophthalmic and Vision Research, and were approved by the Animal Care and Use Committee of the Fourth Military Medical University and the 900th Hospital of Joint Logistic Support Force, PLA. All efforts were made to minimize the number of animals used and their suffering during the whole process.

N-methyl-N-nitrosourea (MNU, CAS NO.: 684–93-5, Aladdin, Shanghai, China) was stored in inert atmosphere at 4 °C and was dissolved in saline containing 0.05% acetic acid immediately before use. Alda-1 (CAS No.: 349438–38-6, TOCRIS, USA), an ALDH2 agonist, was prepared as a stock solution at 0.1 M in dimethyl sulfoxide (DMSO) and was then diluted 1:100 with 50% DMSO in H_2_O (v/v) [20].

### Experimental design

Rats were then randomly divided into the normal (N) group, the model (M) group and the Alda-1 intervention (A) group, with 6 rats in each group. An intraperitoneal injection of 50 mg/kg MNU was applied for the induce of RP in rats from the M and A group. Alda-1, at 5 mg/kg daily, was gavaged to rats from the A group 5 d prior to and for 3 d after the administration of MNU. Meanwhile, rats in the M group received the same volume of vehicle, which contained only the DMSO in H_2_O. Rats from the N group recevied no treatment. Optical coherence tomography (OCT) was used to observe the in-vivo retinal structure of the rats (*n* = 6) in all groups 12 h after the MNU administration. In addition, electroretinogram (ERG) was conducted to assess the retinal function of rats (*n* = 6) at time points of 1 day (D1) and 3 days (D3) after the MNU administration, with OCT performed again immediately after ERG examination. On D3 after the OCT examination, rats (*n* = 6) from all groups were sacrificed by injection of a lethal dose of pentobarbital as an euthanization. The eyes from each rat were immediately harvested,with one eye for retinal histology analysis and the other for protein studies of the underlying molecular mechanisms.

### Experimental technique

#### ERG recording

ERG was performed as previously reported [21]. Briefly, the rats were deeply anaesthetized with an intraperitoneal injection of 1% sodium pentobarbital (3 ml/kg, Sigma-Aldrich, US) and sumianxin II (50 μL for each rat, Jilin Shengda AnimalPharmaceutical Co., Ltd., China) after overnight dark adaptation. Their pupils were dilated with 0.5% tropicamide-phenylephrine ophthalmic solution (Shenyang Xingji Co., Ltd., China). An active electrode was placed on the cornea of each rat. The reference electrode and ground electrode were inserted beneath the skin of the cheek around the tested eye and tail, respectively. Full-field (Ganzfeld) stimulation and a computer system (RETI port, Roland, Germany) were applied to record ERG waveforms. All operations were conducted under dim red light to maximize the retinal sensitivity according to the International Society for Clinical Electrophysiology of Vision (ISCEV) guidelines [22]. Specifically, a brief white flash of 3.0 cd s/m^2^ under a scotopic background was delivered to induce the Dark-adapted 3.0 ERG response. After that, 10 min of light adaptation was administered to achieve stable and reproducible light-adapted ERG and to maximize the response of the cone system and minimize rod input. A brief white flash of 3.0 cd s/m^2^ under a photopic background was delivered to induce the Light-adapted 3.0 ERG response. The b-wave amplitudes of the ERG responses were statistically analysed.

#### OCT scanning

OCT scanning was conducted with the same method as stated previously [23]. Rats, which remained anaesthetized after the ERG recording, were placed on a platform to keep fixed in place. Their pupils were confrimed dilated with the 0.5% tropicamide-phenylephrine ophthalmic solution. The corneas were covered with medical sodium hyaluronate gel (Bausch & Lomb Freda, Jinan, China) and attached to the camera lenses of the 4D-ISOCT Microscope Imaging System (ISOCT, OptoProbe, Canada) to obtain the OCT images. The thickness of the outer retina, namely the length from the inner border of the outer plexiform layer (OPL) to the retinal pigment epithelium (RPE), and the whole retina on both sides of the region 800 μm from the optic nerve were measured in the OCT images using the OCT Image Analysis software (Version 2.0, Optoprobe, Canada).

#### Histological examination

The eyecups from one eye of each rat in all groups were harvested and fixed in 4% paraformaldehyde (in Dulbecco’s phosphate-buffered saline; Mediatech, Inc., Herndon, VA, USA) for 24 h. The tissues were then dehydrated using graded ethanol and were paraffin embedded. Serial sections of 3 μm in thickness were cut vertically through the opitcal nerve. For each eye, 3 sections that included the optic nerve were stained with haematoxylin and eosin (HE, Chengdu, China), images of which were taken using a digital imaging system (DP71, Olympus, Japan). The number of rows of outer nuclear layer (ONL) 800 μm from the optic nerve along the vertically superior-inferior axis were counted at high magnification (× 400).

#### Western blotting

Retinas from the other eye of each rat in all groups were harvested and homogenized on ice in RIPA buffer (Beyotime Biotechnology, China). Aliquot extracts containing equal amounts of protein (30 μg) were electrophoresed, transferred, and probed with primary antibodies against SIRT1 (#ab110304, Abcam, US), ALDH2 (#ab108306, Abcam, US), Glucose-regulated protein 78 (GRP78, #11587–1-AP, Proteintech, US), Poly(ADP-ribose) polymerase 1 (PARP1, #13371–1-AP, Proteintech, US) and GAPDH (#10494–1-AP, Proteintech, US) at 1:1000 dilutions at 4 °C overnight. The membranes were washed and then incubated with goat anti-rabbit HRP-conjugated secondary antibody (#EK020, Zhuangzhi, Xi’an, China) at a 1:10,000 dilution at room temperature for 1 h. An enhanced chemiluminescence system (Thermo Fisher Scientific, US) was used to detect the protein bands, the intensities of which were determined using ImageJ software (Bethesda, MD, USA).

#### Statistical analysis

All data are expressed as the mean ± standard error (S.E.) and were analysed by one-way analysis of variance (ANOVA) followed by Bonferroni test using SPSS software (version 16.0, Chicago, IL, USA). *P* values less than 0.05 were considered statistically significant.

## Results

### Effect of ALDH2 on retinal function

ERG was conducted to investigate the retinal function of rats. The amplitude of the b-wave in the Dark-adapted 3.0 ERG mostly reflects the function of the rods, while that in the Light-adapted 3.0 ERG reflects the function of the cones [22]. On D1, after the MNU injection, the amplitudes of the b-wave in the Dark-adapted and Light-adapted 3.0 ERG in the M group were both significantly lower than those in the N group (*P* = 0.03 for the amplitude in Dark-adapted 3.0 ERG, *P =* 0.008 for the amplitude in Light-adapted 3.0 ERG). The amplitude of the b-wave in the Dark-adapted 3.0 ERG in the A group was significantly higher than that in the M group (*P =* 0.02). However, no statistically significant difference was found in the amplitude of the b-wave in the Light-adapted 3.0 ERG between the M group and A group (*P =* 0.173).

**Fig. 1:**
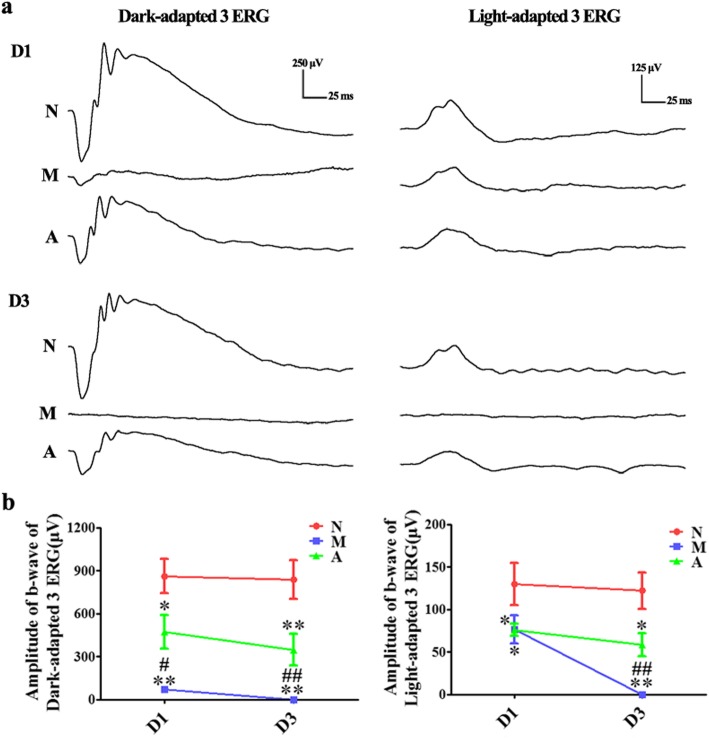
The effect of Alda-1 intervention on retinal function in N-methyl-N-nitrosourea (MNU)-induced retinitis pigmentosa (RP) rats. **a**, **b** Representative waveforms and plots of the b-wave amplitudes in Dark-adapted and Light-adapted 3.0 Electroretinogram (ERG) of rats in all groups. Compared with those of the normal (N) group, the amplitude of ERG, both the Dark-adapted and Light-adapted ERG, in rats of the model (M) group decreased gradually on 1 day and 3days (D1 and D3) after MNU injection, with the ERG waveforms extinguished on D3. The amplitudes in rats in the Alda-1 intervention (A) group also decreased gradually but were significantly higher than those in rats in the M group at each time point (*P <* 0.05). N: the normal group; M: the model group; A: the Alda-1 intervention group. *, **: *P <* 0.05, *P <* 0.01 vs. the N group; #, ##: *P <* 0.05, *P <* 0.01 vs. the M group

On D3, the ERG waveform of the M group was extinguished, with no discernible b-wave in neither the Dark-adapted nor the Light-adapted 3.0 ERG. However, there were still ERG responses from the retinas in the A group, although the amplitudes of the b-wave of the Dark-adapted and Light-adapted 3.0 ERG were both significantly lower than those in the N group (*P* = 0.003 for the amplitude in the Dark-adapted 3.0 ERG, *P =* 0.038 for the amplitude in the Light-adapted 3.0 ERG). (Fig. 1).

### Effect of ALDH2 on retinal structure in vivo

OCT examination was performed to analyse the morphology and thickness of the retinas in vivo. On OCT images, the ONL of the normal retina showed a low reflectivity. At 12 h after the MNU administartion, the area of the ONL in the M and A groups exhibited as a high-reflectivity appearance (shown by the “☆“in the figure), which was obviously different from that of the N group. No statistically significant differences were found in the thickness of the outer retina or the whole retina among all three groups (all *P >* 0.05).

On D1, the high reflectivity of the ONL remained in the M and A groups. The thickness of the outer retina in the M or the A group was significantly lower than that in the N group (all *P <* 0.05). However, there was no statistically significant difference in the thickness of the outer retina between the M and A groups (*P =* 0.237). No statistically significant difference existed in the thickness of the whole retina among the three groups on D1 (all *P >* 0.05).

**Fig. 2:**
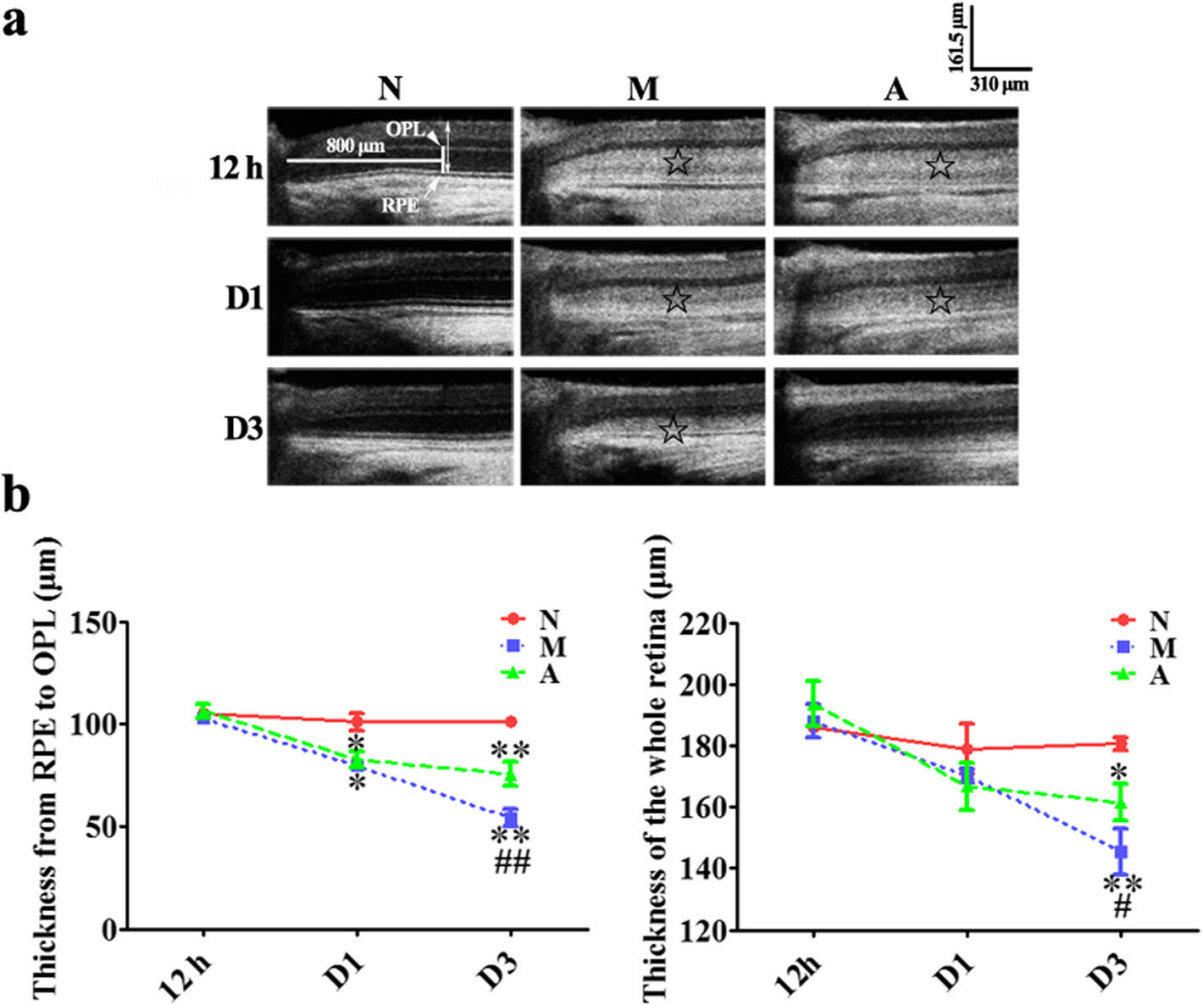
The effect of Alda-1 intervention on the retinal structure of N-methyl-N-nitrosourea (MNU)-induced retinitis pigmentosa (RP) rats in vivo. **a**, **b** Typical retinal optical coherence tomography (OCT) images of rats on 1 day and 3 days (D1 and D3) after MNU administration and statistical analysis of the thickness from the retinal pigment epithelium (RPE) and outer plexiform layer (OPL) (the outer retina) and the whole retina in rats from all groups. MNU induced the convertion of outer nuclear layer (ONL) of rats in the model (M) group from hypo-reflectivity to hyper-reflectivity (shown by the “☆“in the figure) on D1 and D3 after MNU injection, with the thickness of the outer retina gradually decreasing. The ONL in OCT images of rats from the Alda-1 intervention (A) group was reverted into hypo-reflectivity on D3, which resembled that of the normal (N) group. The thickness of the outer retina of the A group was higher than that of the M group on D3. OPL: outer plexiform layer; RPE: retinal pigment epithelium. ON: optic nerve; Scale: 100 μm; ONL: outer nuclear layer. N: the normal group; M: the model group; A: the Alda-1 intervention group. *, **: *P <* 0.05, *P <* 0.01 vs. the N group; #, ##: *P <* 0.05, *P <* 0.01 vs. the M group

On D3, the high reflectivity of the ONL remained in the M group but not in the A group, which exhibited as an low reflectivity. The thickness of the outer retina in the M or the A group was significantly lower than that in the N group (*P* = 0.005 for the difference between the M and N groups, *P* = 0.008 for the difference between the A and N groups). In the A group, the thickness of the outer retina was significantly higher than that of the M group (*P* = 0.03). The thickness of the whole retina in the M or the A group was significantly lower than that of the N group (*P* = 0.007 for the difference between the M and N groups, *P* = 0.03 for the difference between the A and N groups). However, the thickness of the whole retina in the A group was significantly higher than that in the M group (*P* = 0.04). (Fig. 2).

### Effect of ALDH2 on retinal histology

**Fig. 3:**
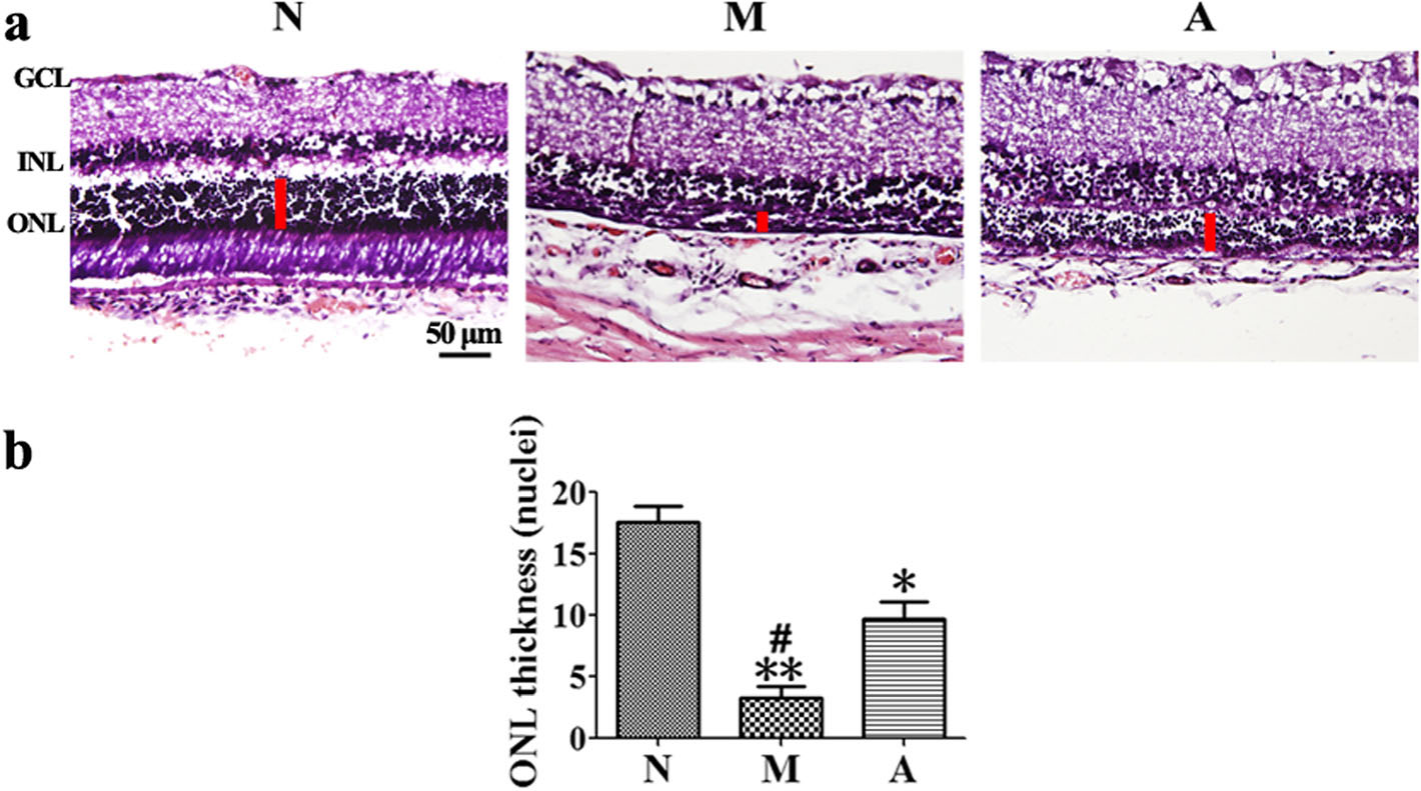
The effect of Alda-1 intervention on retinal histology in N-methyl-N-nitrosourea (MNU)-treated retinas. **a**, **b** Typical haematoxylin and eosin (HE) staining images of retinal sections of rats from all groups and plots of rows of the outer nuclear layer (ONL) 3 days after MNU administration. The number of rows of ONL in the Alda-1 intervention (A) group was lower than that of ONL in the normal (N) group but was higher than that of ONL in the model (M) group, and the differences were statistically significant (*P <* 0.05). ONL: outer nuclear layer; INL: inner nuclear layer; GCL: ganglion cell layer; Scale: 50 μm. N: the normal group; M: the model group; A: the Alda-1 intervention group. *, **: *P <* 0.05, *P <* 0.01 vs. the N group; #, ##: *P <* 0.05, *P <* 0.01 vs. the M group

We performed the HE staining on retinal sections to examine retinal histology. On D3, the number of rows of the ONL in the M group was significantly lower than that in the N group (*P* = 0.001). The number of ONL in the A group was lower than that in the N group (*P* = 0.03), but higher than that in the M group (*P* = 0.02). (Fig. 3).

### Effect of ALDH2 on the expression of some proteins in MNU-induced RP

**Fig. 4:**
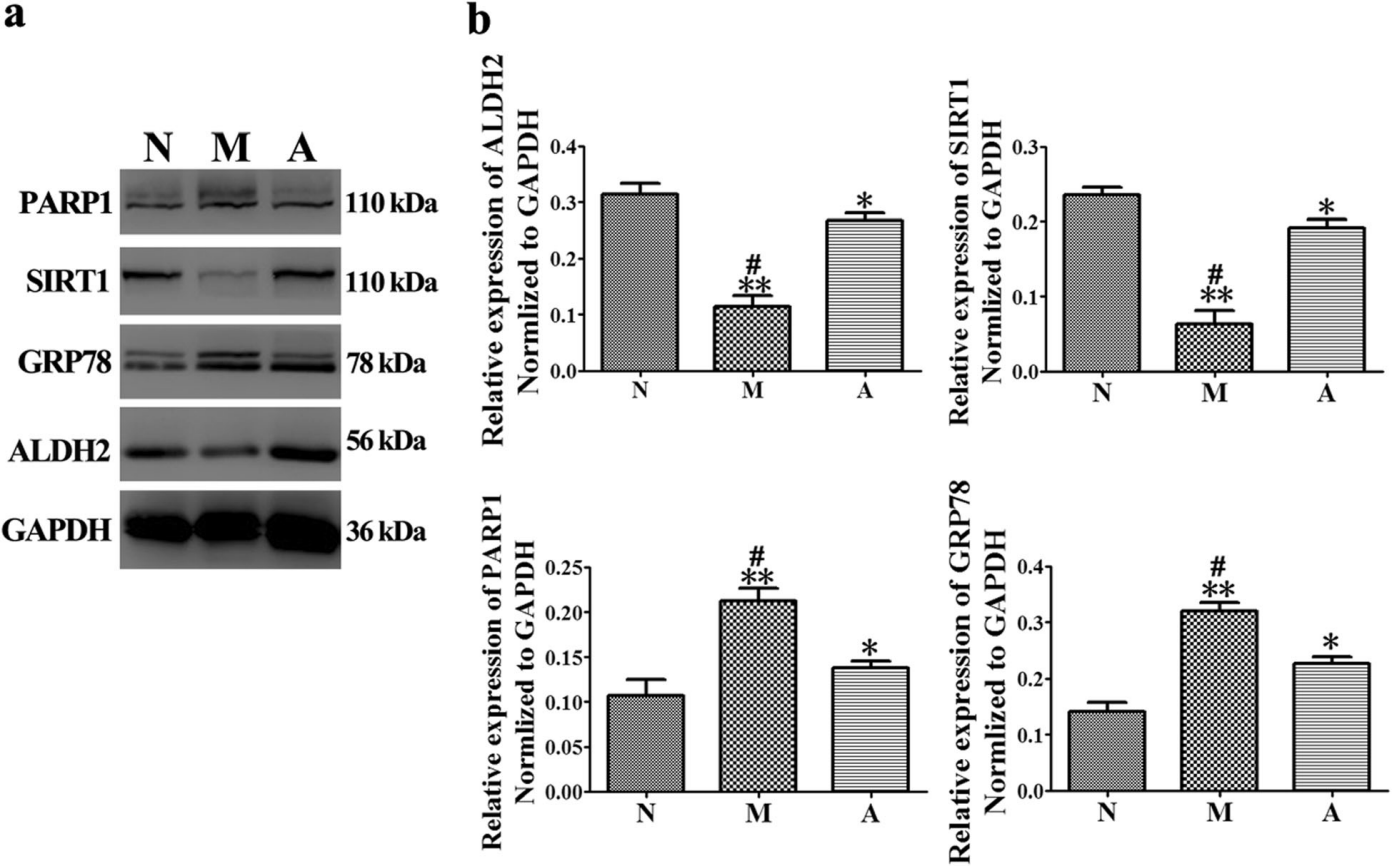
The effect of Alda-1 intervention on the expression levels of some proteins in N-methyl-N-nitrosourea (MNU)-treated retinas. **a**, **b** Representative Western blotting bands and quantitative analysis of ALDH2, SIRT1, GRP78 and PAPR1 expression in retinas from rats in all groups 3 days after MNU administration. In the Alda-1 intervention (A) group, the expression of ALDH2 and SIRT1 in the retinas was higher than that in the model (M) group. In addition, GRP78 and PARP1 expression was lower in the A group than that in the M group, and the differences were statistically significant. N: the normal group; M: the model group; A: the Alda-1 intervention group. *, **: *P <* 0.05, *P <* 0.01 vs. the N group; #, ##: *P <* 0.05, *P <* 0.01 vs. the M group

We chose SIRT1 as a proposed downstream effector of ALDH2, GRP78 as a protein marker of ERS, and PARP1 as a protein marker of apoptosis to investigate the possible related mechanisms of ALDH2-induced protective effects. On D3, the expression levels of ALDH2 and SIRT1 in the retinas of the M group were lower than those in the retinas of the N group (*P* = 0.003 and *P* = 0.005). However, the expression levels of ALDH2 and SIRT1 in the A group were higher than those in the M group (*P* = 0.03 and *P* = 0.02). In the M group, the expression levels of GRP78 and PARP1 were higher than those in the N group (*P* = 0.006 and *P* = 0.004). Meanwhile, the expression levels of GRP78 and PARP1 in the retinas of the A group were significantly lower than those in the retinas of the M group (*P* = 0.03 and *P* = 0.04). (Fig. 4).

## Discussion

In our study, we performed several techniques to investigate the protective effects of Alda-1, the ALDH2 agonist, on MNU-induced RP rats. Together, our results indicate that Alda-1 protects retinal function and structure in MNU-induced RP, of which the mechanisms are at least partly related to the regulation of oxidative stress, ERS and apoptosis.

The MNU-induced animal model of human RP has been studied intensively, with the advantage of an arbitrary modulation of onset and severity with pharmacological approaches [24]. Previous studies show that complete loss of photoreceptors occurs in 1 week after intraperitoneal injection of MNU (Sigma, US) at a dose of 60 mg/kg [25, 26]. Our preliminary studies found that 60 mg/kg MNU from Aladdin (Shanghai, China) induced an RP model on SD rats, of which the ERG waveforms became undetectable in just one or two days. Therefore, we chose a dose of 50 mg/kg MNU for the present study according to studies about different doses of MNU used for RP introduction [27, 28]. The ERG amplitudes became extinguished, and the ONL was almost completely lost three days after administration of 50 mg/kg MNU. The difference in the rate of RP progression might be attributed to the different sources of MNU used (Aladdin vs. Sigma).

High amounts of ALDH2 are found in organs that require high mitochondrial oxidative phosphorylation, such as the liver, heart and brain [29]. However, the expression of ALDH2 in the retina has not been elucidated clearly. Rosemary et al. reported that ALDH2 was principally expressed in Muller glia in the retinas of SD rats by immunohistochemical staining. In addition, they also found a reduction in ALDH1a1 mRNA and protein expression in DR retina, while no such changes were detected for ALDH2 [30]. C. Galbis-esterada et al. found a higher immunoreactive staining of ALDH2 in the inner plexiform membranes, ganglion cells and optic fibre layers as well as in the RPE of the retinas from normal Wistar rats [31]. One of our previous studies showed that ALDH2 was abundantly expressed in the retinas of normal SD rats, mostly in the ONL, while its expression was reduced in DR retinas [19]. The differences in the expression levels of ALDH2 among different studies might be attributed to the different sources of ALDH2 antibodies or the different species of animals used. In the present study, the level of ALDH2 protein was reduced in MNU-treated SD rat retinas, while it was upregulated by Alda-1. Together, the reduced level of ALDH2 in animal models of RP and DR implies that ALDH2 might play a universal role in the pathogenesis of retinal diseases.

Our ERG data showed that the deterioration of retinal function in both rod and cone responses was delayed by Alda-1 treatment, although the protective effect on the cone response did not show a statistical difference on D1 after Alda-1 administration. The cone cell is affected later than the rod cell in the model of MNU-induced RP, which is similar to that of human RP [32]. In accordance with other reports [33, 34], the reduced amplitude of the cone response in ERG in our study was significantly lower than that of rod response. Thus, the range of change in amplitude of the cone response in ERG on D1 might be too small to demonstrate the Alda-1-induced protection, which was more obvious in rod response.

For retinal morphology, MNU induced the convertion of ONL from hypo-reflectivity to hyper-reflectivity as seen on the OCT images, which might be related to the apoptosis of photoreceptors [35, 36]. This phenomenon has also been found in light-induced retinopathy [37] and in inherited RP mice [38]. Our data revealed the protection of retinal morphology by Alda-1, as the ONL returned to a hyporeflective status on D3. However, the thickness of the outer retina and the number of ONL with Alda-1 intervention were still reduced compared to those in the N group. The present data demonstrated that Alda-1 (5 mg/kg by gavage) could only reduce but not completely prevent the damage of retinal function and morphology in RP induced by 50 mg/kg MNU. The gavage dose of Alda-1 was adjusted according to other studies of Alda-1 administration [39, 40]. As described previously, the RP model applied in our study might still be too severe and the course of Alda-1 administration is only three days. Therefore, we speculate that adjusting the dose or delivery of Alda-1 and reducing the dose of MNU for RP introduction might attain better protective effects of Alda-1 on retinas.

The loss of photoreceptors in RP has been commonly attributed to the apoptosis of photoreceptors [2, 41]. PARP1, an endogenous nuclear enzyme catalysing the poly(ADP-ribosyl)ation, is commonly considered as an important biomarker of apoptosis [42]. PARP1 has been implicated in the development of retinal degenerative diseases, and downregulated PARP1 expression might play a role in the preservation of photoreceptors [43, 44]. In our study, the survival of photoreceptors was accompanied by decreased expression of PAPR1 after Alda-1 administration. It seems that ALDH2 may modulate PARP1 expression, leading to the alleviation of photoreceptor apoptosis in RP. To our knowledge, the present study presents the first demonstration of the impact of ALDH2 on PARP1 expression.

The role of SIRT1, an NAD^+^-dependent histone deacetylase, in the progress and protection of retinal degeneration has been reported in great details in several disease models [45, 46]. Jaliffa, C et al. found that the pathologic expression pattern of SIRT1 was correlated with initial retinal degeneration in rd10 mice [47]. Qi et al. systematically proved the pivotal role of SIRT1 in the alleviation of light-induced photoreceptor degeneration by administration of a SIRT1 agonist and antagonist [48]. We previously showed that SIRT1 might be the reason for the amelioration of MNU-induced RP by hydrogen-rich saline, a newly-found antioxidant [49]. Our present data demonstrated that the upregulation of ALDH2 by Alda-1 was closely accompanied by the increased expression of SIRT1 in the amelioration of photoreceptor loss. We also detected the upregulation of SIRT1 expression in the protection against DR by ALDH2 activation in a previous study [19]. Besides, ALDH2 has been demonstrated in another research to mediate the protection of chronic ethanol against endothelial senescence through the SIRT1 pathway in vitro [50]. In the model of myocardial ischaemia/reperfusion (MI/R) injury, cardiac-specific ALDH2 upregulation by viral gene delivery significantly ameliorated chronic-pain-induced SIRT1 carbonylative inactivation and decreased MI/R injury [51]. These findings together indicate that SIRT1 might be a downstream regulator of ALDH2-induced effects.

The involvement of the ERS has been increasingly recognized as a contributor in a variety of diseases, including glaucoma [52], DR [53], AMD [54] and RP [55]. GRP78, a protein chaperone, was chosen as the ERS marker in the present experiment [56]. Our results showed that GRP78 was actively involved in the progression of MNU-induced RP, which was in accordance with other investigations [57]. Moreover, Alda-1 intervention decreased the expression of GRP78 to some extent, indicating the possibility that ALDH2 relieves the ERS during RP. Similarly, the alleviation of the ERS by ALDH2 has also been found in other conditions. In a mouse model of alcohol-induced liver injury, ALDH2 was able to render protection by reducing the expression of ERS markers, IRE1alpha and p-PERK, while there was no significant effect on the expression of ATF6 [58]. In ERS-mediated myocardial cell injury induced by tunicamycin, ALDH2 administration was argued to ameliorate the injury of myocardial cells mainly by regulating the PERK pathway [59].

In our study, the variation trends of ALDH2 and SIRT1 were highly consistent, in line with the downregulation of the ERS and apoptosis markers. Downregulation of SIRT1 has been suggested to cause a vascular/endothelial ERS [60]. The SIRT1 pathway has also been shown to play an essential role in the exenatide-induced alleviation of the lipid-induced ERS and hepatic steatosis [61]. In addition, Kang, X. et al. reported that SIRT1 inhibition mainly induced the PERK-eIF-2alpha-CHOP axis of the ERS response in the growth-plate chondrocytes, while the EX527 or SIRT1 siRNA-mediated inhibition of metatarsal growth and growth-plate chondrogenesis was partly neutralized by a chemical chaperone that attenuates the ERS [62]. In terms of the relationship between SIRT1 and ERS, we supposed that the protective effects of ALDH2 against photoreceptor apoptosis in RP might be mediated through the SIRT1/ERS pathway, which requires further confirmation in future study.

There were some limitations in our studies, which are listed below: 1. The dose-dependent effect of Alda-1 was not observed. 2. The observation period of the Alda-1’s effect on MNU-induced RP was not long enough. The checkpoint in the present study was only 1 and 3 days after MNU administration. 3. The control group in our present research included only rats with vehicle administration after MNU injection. A control group consisting of Alda-1 alone should be included in the future study as to explore whether Alda-1 alone would affect the normal retinas. 4. The levels of aldehyde were not measured in our study. It would be more complicated to measure the aldehyde levels in the rat retina of all groups, which could verify the protective effect of Alda-1 on the retina. In the near future, we would design experiments to thoroughly evaluate the effects of various doses and different delivery routes of the ALDH2 agonist and the ALDH2 inhibitor. These would help to further explore the molecular mechanisms of ALDH2-induced protection on retinal function and structure.

## Conclusions

In conclusion, Alda-1, the ALDH2 angonist, ameliorates photoreceptors from degeneration in MNU-induced RP retinas, which is possibly associated with modulation of SIRT1/ERS-related apoptosis. Our study reveals that ALDH2 is a possible target in photoreceptor apoptosis, which may increase the understanding of ALDH2 as a potential target in new therapeutic approaches for RP. Future studies are necessary to clarify the details of the pathways in which ALDH2 provides protection against RP.

## Data Availability

The datasets used and analysed in the present study are available from the corresponding author on reasonable request.

## Abbreviations

4-HNE: 4-hydroxy-2-nonenal
Alda-1: N-[1,3-benzodioxol-5-ylmethyl]-2,6-dichlorobenzamide
ALDH2: Acetaldehyde dehydrogenase 2
AMD: Age-related macular degeneration
ANOVA: One-way analysis of variance
ARVO: Association for Research in Vision and Ophthalmology
DMSO: Dimethyl sulfoxide
DR: Diabetic retinopathy
ERG: Electroretinograms
ERS: Endoplasmic reticulum stress
GRP78: Glucose-regulated protein 78
ISCEV: International Society for Clinical Electrophysiology of Vision
JNK: C-Jun NH2-terminal kinase
LPO: Lipid peroxidation
MDA: Malonyldialdehyde dimetyl 1,1,3,3 tetramethoxypropanol
MI/R: Myocardial ischaemia/reperfusion
MNU: N-methyl-N-nitrosourea
OCT: Optical coherence tomography
ONL: Outer nuclear layer
OPL: Outer plexiform layer
PARP1: Poly(ADP-ribose) polymerase 1
ROS: Reactive oxygen species
RP: Retinitis pigmentosa
RPE: Retinal pigment epithelium
SD: Prague-Dawley
SIRT1: Silent information regulator-1
